# Group phenotypic composition in cancer

**DOI:** 10.7554/eLife.63518

**Published:** 2021-03-30

**Authors:** Jean-Pascal Capp, James DeGregori, Aurora M Nedelcu, Antoine M Dujon, Justine Boutry, Pascal Pujol, Catherine Alix-Panabières, Rodrigo Hamede, Benjamin Roche, Beata Ujvari, Andriy Marusyk, Robert Gatenby, Frédéric Thomas

**Affiliations:** 1 Toulouse Biotechnology Institute, University of Toulouse, INSA, CNRS, INRAEToulouseFrance; 2 Department of Biochemistry and Molecular Genetics, University of Colorado Anschutz Medical CampusAuroraUnited States; 3 Department of Biology, University of New Brunswick, FrederictonNew BrunswickCanada; 4 CREEC/CANECEV, MIVEGEC (CREES), University of Montpellier, CNRS, IRDMontpellierFrance; 5 Centre for Integrative Ecology, School of Life and Environmental Sciences, Deakin UniversityGeelongAustralia; 6 Laboratory of Rare Human Circulating Cells (LCCRH), University Medical Centre of MontpellierMontpellierFrance; 7 School of Natural Sciences, University of TasmaniaHobartAustralia; 8 Department of Cancer Physiology, H Lee Moffitt Cancer Center and Research InstituteTampaUnited States; Vanderbilt UniversityUnited States; Pennsylvania State UniversityUnited States

**Keywords:** cancer biology, ecology, medicine, evolutionary biology

## Abstract

Although individual cancer cells are generally considered the Darwinian units of selection in malignant populations, they frequently act as members of groups where fitness of the group cannot be reduced to the average fitness of individual group members. A growing body of studies reveals limitations of reductionist approaches to explaining biological and clinical observations. For example, induction of angiogenesis, inhibition of the immune system, and niche engineering through environmental acidification and/or remodeling of extracellular matrix cannot be achieved by single tumor cells and require collective actions of groups of cells. Success or failure of such group activities depends on the phenotypic makeup of the individual group members. Conversely, these group activities affect the fitness of individual members of the group, ultimately affecting the composition of the group. This phenomenon, where phenotypic makeup of individual group members impacts the fitness of both members and groups, has been captured in the term ‘group phenotypic composition’ (GPC). We provide examples where considerations of GPC could help in understanding the evolution and clinical progression of cancers and argue that use of the GPC framework can facilitate new insights into cancer biology and assist with the development of new therapeutic strategies.

## Introduction

Primary cancers often display high levels of phenotypic and genetic intratumor heterogeneity (ITH). Whereas the mutational and epigenetic mechanisms that generate this heterogeneity are relatively well understood, mechanisms responsible for maintaining this heterogeneity in cancer cell populations, as well as the impact of ITH on clinically important properties of the disease, are less clear. Yet, high levels of ITH have been linked to more aggressive tumor behavior, resistance to therapies, and overall poor prognosis (Marusyk and Polyak, 2010; Meacham and Morrison, 2013; McGranahan and Swanton, 2015; Morris et al., 2016; McGranahan et al., 2017; Dagogo-Jack and Shaw, 2018; Hausser and Alon, 2020; Marusyk et al., 2020). Thus, a deeper understanding of ITH at different stages during cancer progression might result in novel therapeutic strategies directed at altering tumor composition towards decreased malignancy.

ITH can be subdivided into micro-heterogeneity (i.e., cell–cell variability) as well as macro-heterogeneity (i.e., heterogeneous subgroups), yielding a co-existence of either independent genetic subclonal lineages or metastable phenotypic subgroups (Merlo et al., 2006). Despite the rapidly growing interest in assessing ITH, the complex eco-evolutionary dynamics between cancer cells and their environment, the balance between heritable and non-heritable cellular traits, as well as the resulting effects on global tumor behavior remain poorly understood.

In particular, stable co-existence of distinct subpopulations or subclones within tumor cell populations that follow Darwinian dynamics appears to be counterintuitive. Indeed, the maintenance of stable subpopulations with heritable phenotypes within the same tumor indicates the presence of multiple niches producing complex patterns of co-existence, although weak stabilizing selection and drift may also contribute to phenotypic variability. In turn, the co-existence of different subpopulations can profoundly influence global tumor behavior such that elimination of one subpopulation can induce tumor regression or necrotic collapse (Inda et al., 2010; Marusyk et al., 2014). This co-existence can be based on ‘cooperation’ (or ‘mutualism’ in ecological terms) between distinct subpopulations (Merlo et al., 2006; Axelrod et al., 2006; Tabassum and Polyak, 2015; Zhou et al., 2017; Martín-Pardillos et al., 2019). Synergistic interactions between two subpopulations of cancer cells may also permit metastatic spread (Marusyk et al., 2014; Calbo et al., 2011; Neelakantan et al., 2017; Janiszewska et al., 2019). Importantly, reversible phenotype switching seems to have a prominent role in the dynamics of interactions between diverse subclones (Chapman et al., 2014), suggesting that the intratumor phenotypic heterogeneity arising from non-genetic variability (i.e., phenotypic plasticity) permits a rapid, reversible strategy for cancer cells to optimally respond to changing environmental conditions and population fluctuations. In addition, populations of tumor cells also exhibit Allee effects (i.e., positive correlation between population density and individual fitness) in which the proliferation rate paradoxically increases with population size, which promotes aggressive tumor growth (see for instance Korolev et al., 2014; Böttger et al., 2015; Sewalt et al., 2016; Johnson et al., 2019). However, at the moment, investigations on these interactions are limited to few subpopulations (Marusyk et al., 2014; Calbo et al., 2011), molecular interactions (Inda et al., 2010; Vinci et al., 2018; Wu et al., 2010; Cleary et al., 2014), and cancer hallmarks (Chapman et al., 2014; Wagenblast et al., 2015). Moreover, these examples represent a snapshot of tumor progression, while the interactions between subpopulations can be highly dynamic and change over time. To capture these dynamics, we suggest applying an ecological concept termed ‘group phenotypic composition’ (GPC), to understand the ecology/evolutionary dynamics of cancer.

In nature, the function and stability of a group is dependent on the characteristics of the constituent individuals (and/or subgroups composed of individuals sharing common features) and their interactions, which are always in an evolutionary state of flux. Tumors have already been described as composed of functional compartments with a division of labor among these compartments (Grunewald et al., 2011; Hausser et al., 2019) and conceived as a whole consortium of cooperating malignant clones (Ramón Y Cajal et al., 2017). Other studies suggested that phenotypic groups can be defined among tumoral cells on the basis of common expression of phenotypic markers, even if these phenotypic clusters can display genetic and epigenetic heterogeneity (see Box 1). Here, we propose to extend the concept of GPC toward tumor cell populations and investigate how the phenotypic composition of tumors can impact cancer progression and treatment.

Box 1. Intratumoral phenotypic clustering despite genetic and epigenetic diversity.

Cancer cells can reversibly and stochastically transit between states that differ in their competence to contribute to tumor progression (Gupta et al., 2011). These stochastic cell-state transitions can not only generate cells with the properties of cancer stem cells (CSC), but also make cells transdifferentiate without acquisition of a stem-like state. This cancer cell plasticity can contribute to tumor initiation, progression, and therapeutic resistance (Capp, 2019; Gupta et al., 2011; Pisco and Huang, 2015) and is expected to greatly contribute to cell-to-cell heterogeneity in cancer (Meacham and Morrison, 2013; Marusyk et al., 2020). For instance, transdifferentiation can be a major driver of drug resistance and metastasis through a mechanism that ultimately relies on epigenetic mechanisms (Sharma et al., 2018). The ability to transit, especially toward a CSC state, is dependent on either intracellular stochastic events or microenvironmental perturbations (Nguyen et al., 2012; Beck and Blanpain, 2013). Indeed, various phenomena such as epigenetic regulation, stochastic gene expression, or variability in the microenvironment can contribute to phenotypic diversification (Marusyk et al., 2012). Among them, histone modifications were shown to confer lineage plasticity in cancer (Pastore et al., 2019; Liau et al., 2017; Flavahan et al., 2017). For instance, intratumoral epigenetic diversity allows leukemic cells to stochastically activate alternative gene regulatory programs, facilitating the emergence of novel cell states, ultimately enabling tumor growth and drug tolerance (Pastore et al., 2019). The strong increase in entropy of the cancer epigenome appears to increase stochasticity of gene expression at higher levels than normal stem cells because of a less organized and less stable chromatin structure (Jenkinson et al., 2017), thus greatly impacting phenotypic variability.

Interestingly, when a subpopulation is purified for a given phenotypic state, a return toward equilibrium proportions is then observed over time (Gupta et al., 2011), suggesting that such phenotypic transitions occur regardless of the cell’s genetic content. A pioneering study already showed that the distinct cell populations and the underlying transcriptional heterogeneity observed among single human colon cancer cells were not due to underlying genetic heterogeneity, as injection of single CSC into immune-deficient mice gave rise to monoclonal tumors as heterogeneous as the parental one (Dalerba et al., 2011). To generalize these observations, it is now recognized that phenotypic clusters (groups of cells expressing the same phenotypic markers) exist in vivo among tumor cells (Wagner et al., 2019; Jackson et al., 2020), despite divergent genomes, epigenomes, and clonal origins. The same functional clustering has been suggested for breast cancers despite high intertumoral heterogeneity: diverse genetic and epigenetic alterations converge phenotypically into four main breast cancer classes (Cancer Genome Atlas Network, 2012).

Innovative methods such as single-cell mass cytometry allow the detection of phenotypic clusters among single tumoral cells despite extensive heterogeneity in genetic and epigenetic content. For instance, a consensus clustering approach allowed hierarchical clustering of 45 epithelial clusters in breast cancers based on normalized epithelial cell marker expression (Wagner et al., 2019). Recently, by designing an imaging mass cytometry panel specific to breast histology, it was possible to quantify spatial inter- and intratumor phenotypic heterogeneity at the single-cell level among 281 tumors using 35 antibodies (Jackson et al., 2020). This strategy allowed mapping the cellular spatial organization of these tumors and to observe variable structures and cellular densities. It also revealed relationships between cellular phenotype, tissue organization, and clinical outcome. In summary, both heterogeneous tumors with multiple phenotypically pure communities and homogeneous tumors with one epithelial sheet or with similar communities of different sizes were observed, the former being associated with poorer outcomes. The authors concluded that studying how spatial and phenotypic tissue features influence disease outcome might be medically relevant.

### GPC in the animal kingdom and its relevance to cancer

Group living is a widespread phenomenon within the animal kingdom and has attracted considerable attention among evolutionary ecologists (Farine et al., 2015; Koenig et al., 2020). Recent advances in this area have highlighted that GPC is a crucial parameter to consider. Farine et al., 2015 define GPC as “any descriptor of the types of phenotypes found within a group. Examples include the average body size of individuals, the variation in male color, or the aggressiveness level of the most aggressive individual in the group. In this framework, ‘groups’ can represent many aspects of the social environments of individuals, including breeding units, social networks, neighborhoods, populations, and communities.” Members in any group, be it individuals in populations or species in communities, express some phenotypic variation at several levels (e.g., activity, behavior, morphology, life-history traits, etc.). The heterogeneous composition of groups provides them with different properties, due to the different average phenotypes, the presence of keystone individuals, and/or the variability in phenotypes etc., that can in return influence group-level outcomes (e.g., foraging success, mating system, predation risk, evolvability). While theory on the implications of GPC is still in its infancy, the concept proved relevant to understand many aspects of group-level dynamics and self-organization (Farine et al., 2015). In addition, selective pressures arising from GPC properties can influence the evolutionary trajectory of individual phenotypes. For instance, large groups composed of individuals with similar phenotypes may benefit from a reduced predation pressure because of the confusion effect that the lack of prey ‘oddity’ generates on predators (Landeau and Terborgh, 1986; Krakauer, 2004). In prey species such as the cladoceran *Daphnia magna* for which predation risk (by the three-spine stickleback *Gasterosteus aculeatus*) is higher on larger individuals (i.e., more profitable preys) and confusion effects operate, some conflicts between individuals with different phenotypes might emerge: both large and small individuals benefit from joining groups composed of large individuals, but the presence of small individuals enhances the predation risk experienced by large ones (Rodgers et al., 2015). Selection from GPC can thus alter the covariance between individual and group phenotypes through the removal of particular phenotypes within generations. In the *Daphnia*/stickleback system, excluding or avoiding small individuals would be beneficial for large individuals, but it also comes with (energetic) costs, leading to mixed groups in the wild. This example illustrates the kind of complex and reciprocal links that may exist between individuals’ phenotypes and the GPC’s properties. More generally, individual heterogeneity and resulting GPCs result in multiple hierarchical effects that affect collective behavior, from within-group positioning, group coherence, leadership, and collective decision making to group functioning, fission–fusion dynamics, and among group assortment (see Jolles et al., 2020 for a recent review).

**Figure 1.**
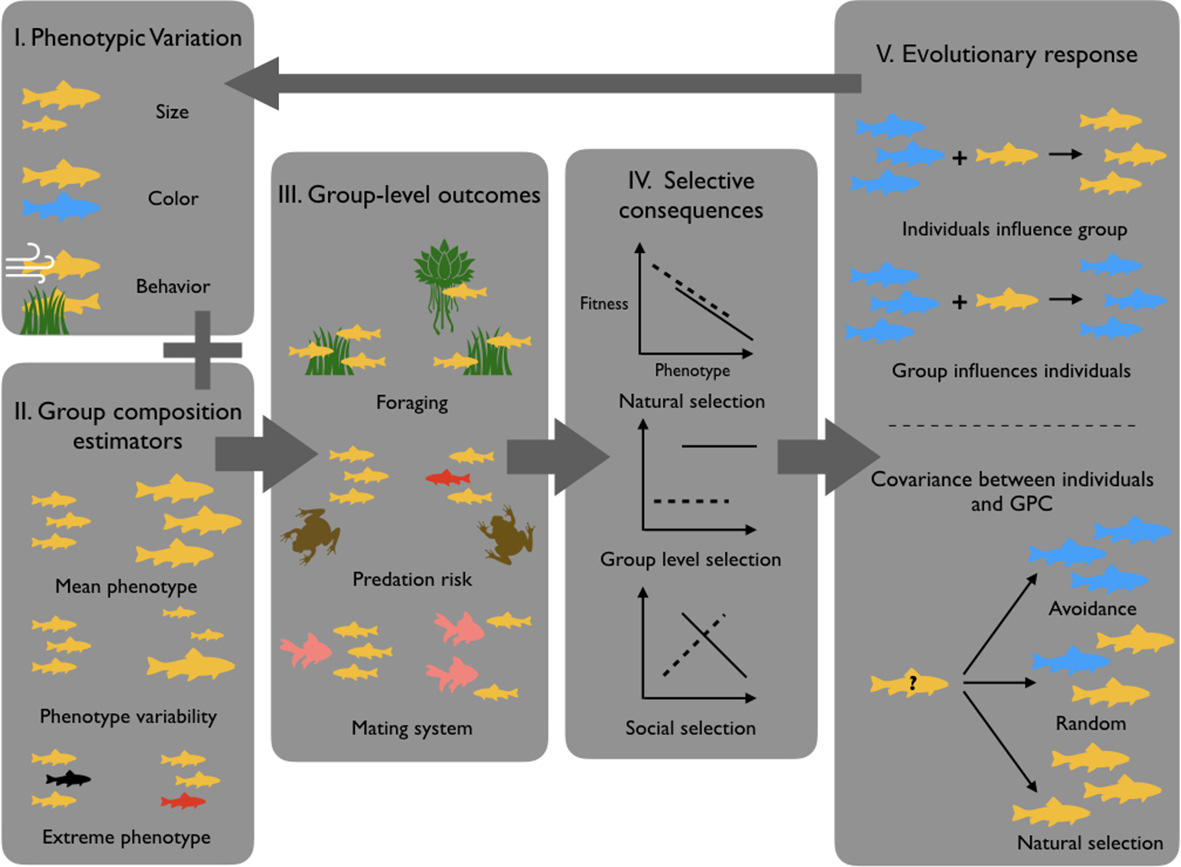
GPC and its ecological/evolutionary implications. Because individuals show significant phenotypic variation (**I**), groups may vary in their GPC depending on which individuals constitute the group (**II**). This may in return influence group-level outcomes (**III**), which as a consequence differentially impacts individual fitness (**IV**). (**V**) GPC then drives different evolutionary responses, like for instance decisions to join or leave particular groups or phenotypic plasticity in response to GPC. These phenomena can then influence the distribution of phenotypes in subsequent generations (figure modified from Farine et al., 2015).

Farine et al., 2015 provided a review with many examples as well as a conceptual framework (Figure 1) allowing one to understand and predict when GPC should have important selective consequences. GPC in the animal kingdom has both pervasive consequences for individual fitness and fascinating evolutionary implications, with potential to contribute to many classical questions in ecology and evolution.

In so far as tumors are heterogeneous subgroups of cells that interact among each other and with other livings entities, for example host normal cells with the microbiota, it is theoretically possible to examine them within the framework of the GPC. Noticeable ecological and evolutionary singularities however exist, given that most cancers are not transmissible and hence cannot evolve for longer periods than the life expectancy of their host, and because tumors are usually not in competition inside the hosts (except in transmissible tumors like DFTD and DFT2 in Tasmanian devils, see Thomas et al., 2018). Despite these differences for the majority of other cancers, there is still predation of malignant cells by the immune system and competition between cells and/or between clusters of cells within tumors. The extrapolation of Farine et al.’s definition of GPC to cancer cannot totally rely on the same descriptors because groups are composed here of cells, not animals, and thus cells’ specificities need to be considered. For example, phenotypes in animal models that depend on a high level of mobility and rapid communication within the group might not translate well because cancer cells move slowly and communicate slowly by diffusible factors. Similarly, the high mutagenic rate of cancer cells is likely to impact group behavior, while the slow rate of mutation seen in whole organisms is unlikely to significantly alter group functioning. The extent to which mechanisms driving evolution or heterogeneity in cancers might have potentially useful analogs in other group situations still needs to be explored. For instance, promising aspects could include (1) the importance of epigenetic modulation of gene expression in cancer cells vs the Baldwin effect (process by which plasticity facilitates evolution) in whole organisms, (2) how might the asexual nature of cancer cell reproduction influence individual–group evolutionary relationships relative to sexually reproductive individuals in other ecosystems, and (3) what phenotypes in cancer need to be quantified to be able to produce group selection models that are useful for describing progression or influencing clinical practice. Thus, more investigations are required at the moment to fully appreciate what is unique about cancer that will define its individual–group relationships differently from other ecosystems (see also Box 1).

## Group phenotypic composition in the context of cancer

It is traditionally assumed that individual cells are Darwinian units of selection in cancers (Greaves, 2013), as they possess the required prerequisites that include (1) phenotypic variation, (2) differential fitness co-variant with a phenotypic trait, and (3) heritability (Lewontin, 1970). However, some aspects of cancer biology, such as promotion of angiogenesis, evasion of immune predation, or environmental engineering involving remodeling of extracellular matrix require group action. Yet, the evolutionary dynamics that govern these groups (including the level that selection acts on) have been poorly explored (Greaves, 2013; Bertolaso and Dieli, 2017; Lean and Plutynski, 2016). Furthermore, the ecological factors and evolutionary forces shaping these interactions as well as the outcomes of these processes are likely to change during cancer progression. Nevertheless, (1) how the phenotypic composition of a group (i.e., its average phenotype or phenotypic variance) can affect ecological and social processes (e.g., niche construction, competition, cooperation) and (2) how multi-level selection can drive phenotypic covariance among interacting cancer cells are not understood. Following Farine et al., 2015 framework, we propose that addressing the role of GPC as both an agent of selection shaping individual fitness and an emergent property of the individual phenotypes can help understand cancer progression and predict ways that can alter GPC to affect both individual- and group-level outcomes.

How do we define GPC in cancers? We envision a group to (1) be limited to cells that share a set of driver (epi)mutations (but which could subsequently diverge genetically and epigenetically and produce cooperating subgroups each composed of cells sharing at least a common phenotype, see also Box 1), (2) be spatially restricted, given that there are physical limitations to cooperation and competition, and (3) have interactions that are highly dependent on microenvironmental context (considering both other competing normal and malignant cells). Note that we are not including normal cells as part of the malignant group, although its GPC and collective properties will be highly influenced by the interactions with normal cells within the tumor microenvironment.

Soon after initiation, a tumor constitutes a small group, but as it grows, new subgroups can be distinguished, based on new spatial structures and interactions, and its phenotypic composition is likely to change. Whereas specific characteristics, sufficient to define individual subgroups within the GPC still need to be rigorously defined, we envision useful GPC descriptors to include average proliferation rate, degree of genetic or phenotypic heterogeneity, proportion of cells with distinct differentiated states (such as mesenchymal and epithelial for carcinomas), type (luminal and basal, for breast tumors), amount and composition of extracellular matrix, type and numbers of non-malignant cells, proportion of drug-resistant cells, etc.

GPC can be dynamic both in terms of number, types, and proportion of subgroups, and codependency levels between subgroups. For instance, the emergence of macroscopic tumors is dependent on the presence of a threshold number of tumor cells that produce angiogenic factors (Folkman and Klagsbrun, 1987). These factors represent ‘public goods’ that can increase the fitness of all cells, which will ultimately alter the tumor composition. Another example of a public good is the enzyme indoleamine-pyrrole 2,3-dioxygenase, which catalyzes the degradation of tryptophan in the cell’s environment leading to the inhibition of cytotoxic immune responses thereby improving survival of both producing and non-producing cells (Munn and Mellor, 2016). In groups that expand due to sufficiently high proportion of producers, cells that do not produce angiogenic factors (i.e., ‘cheaters’ that rip the benefits of the common goods without incurring the potential cost associated with their production) can potentially outcompete the producers leading to a collapse of the vascular network (Epstein et al., 2017; Gravenmier et al., 2018).

**Figure 2.**
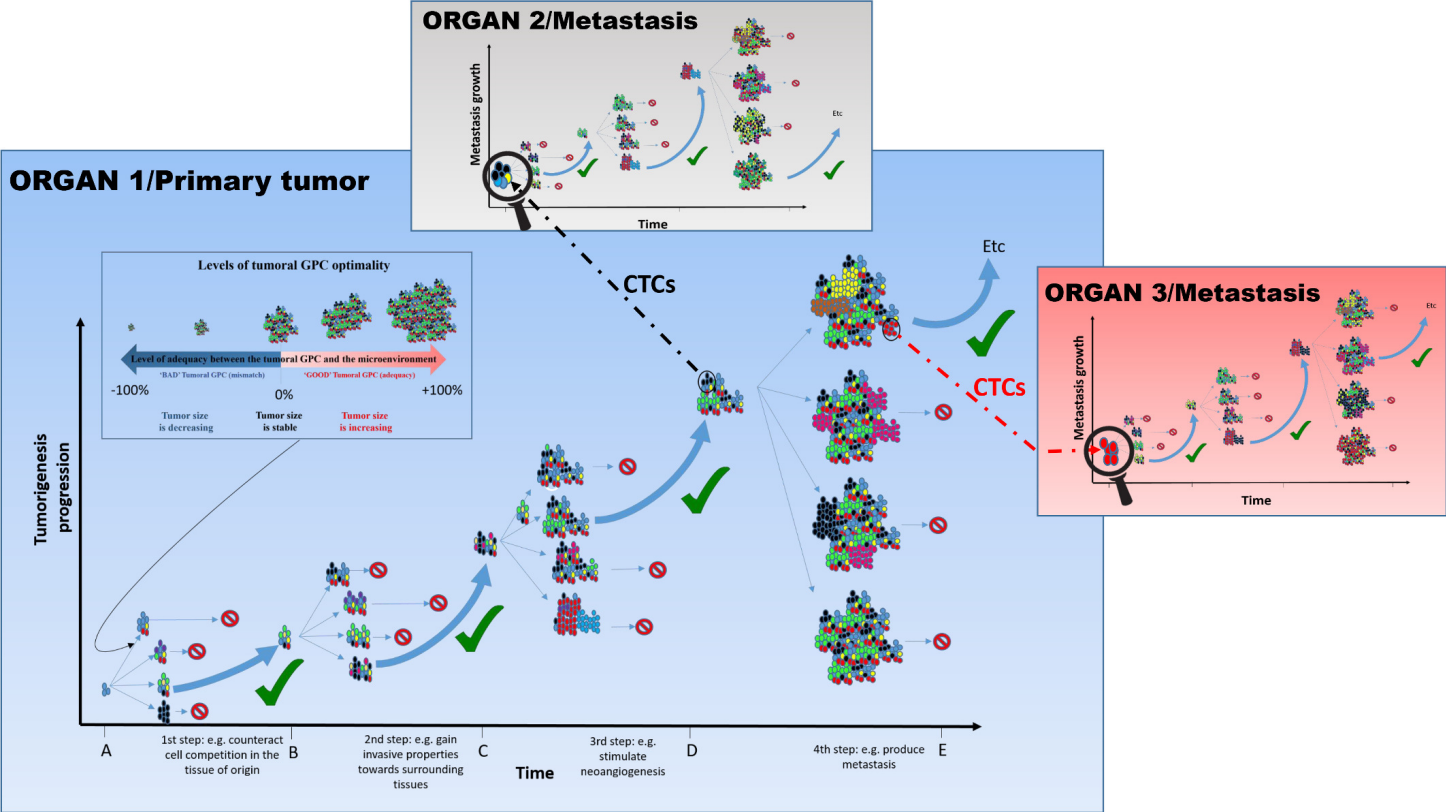
The tumoral GPC framework. Cancer cell proliferation and mutation in a tumor can produce different possibilities of tumoral GPCs, depending on the relative fitness of cancer cells at a given time (different colored cells represent distinct evolutionary lineages). Depending on the resulting tumoral GPC, the tumor, viewed as the habitat in which malignant cells live and evolve, possesses specific group properties (e.g., quality of the vascular network, level of immunogenicity, etc.). These properties can, in return, affect (positively or negatively) cell fitness, and hence tumor growth. In the absence of selection at the group level, or of an encoded tumorigenesis program, it is potentially frequent that conditions that increase cell-level fitness of one clonal lineage can result in a non-optimal or even detrimental tumoral GPCs, which can slow down or stop tumor growth, and/or even induce its size reduction. Since tumors of different sizes have different requirements and interactions with their changing microenvironment, tumoral GPC varies with the tumor stages and the microenvironment; that is, there is no single optimal tumoral GPC that is maintained throughout cancer progression. Only tumors that achieve a successful/adequate tumoral GPC at each step of tumorigenesis will evolve into metastatic tumors. The tumors that fail to generate an adequate tumoral GPC at a given step do not necessarily disappear, they just do not continue to expand. Those that produce an inadequate tumoral GPC, for instance leading to higher immunogenicity, may become reduced in size and even disappear. This hypothesis can explain why we can develop many neoplasms in the body, but the majority of them never grow until the metastatic stage or even regress (Folkman and Kalluri, 2004). Circulating tumor cells (CTCs), especially clusters that can be either homogeneous (organ 3) or heterogeneous (organ 2), can disseminate and initiate metastasis where a novel process of diversification is required so as to harbor the right GPC in a given organ and develop into advanced metastasis.

Tumor growth and progression are associated with changes in microenvironmental contexts in space and time (such as acidification, inflammatory response, hypoxia etc.). In turn, these contextual changes would be expected to impact fitness of subgroups of tumor cells. Thus, a GPC that is highly ‘optimal’ (reflected in proliferation and death of subgroup members, as well as in expansion of the group) during the early stages of tumorigenesis (e.g., composed of highly proliferative cells) may not be optimal in a large tumor (which requires vasculature) (Losi et al., 2005; Lee et al., 2015; Nguyen et al., 2016; Saito et al., 2018; Figure 2; steps 2 and 3). Similarly, the transition to a metastatic tumor relies on a different ‘optimal’ GPC (e.g., including a higher proportion of ‘aggressive’ cells) that reflects changes in the tumor microenvironment (Figure 2; step 4). This change in GPC might involve epigenetic changes as well as phenotypic plastic responses in response to microenvironmental and/or paracrine signals, from both cancer and non-cancer cells. In addition to these spatio-temporal contextual changes within dynamic tumors, the differences in the overall context prior to cancer initiation, such as genetic makeup, sex, hormonal environment, aging, are expected to alter the match between GPC and its impact on the fitness of the group (Laconi et al., 2020; Vibishan and Watve, 2020).

Thus, tumoral GPC, and its relationships with the fitness of individual malignant cells in different ecological contexts, may represent a crucial, previously unexplored aspect that influences evolutionary dynamics within tumor cell populations. In this context, GPC will influence selection pressures experienced by individual tumor cells, which will influence individual cell-level outcomes. As the relative proportion and type of interactions among subgroups of tumor cells vary in response to changing microenvironments, tumoral GPCs will also change resulting in either new optimal tumoral GPCs or, potentially, catastrophic imbalances producing tumor extinction (Figure 2; in this case, tumoral GPC can be seen as the target of selection). As discussed further below, it might be possible to affect GPC to achieve desirable therapeutic outcomes, including tumor stasis or extinction.

The GPC concept might also be applicable to circulating tumor cells (CTCs) (see also Box 2). Individual metastatic tumors can be viewed as reproducing groups (Lean and Plutynski, 2016), where CTCs are seeds of successful groups that travel through blood and lymphatic vasculature to reach distant fertile ‘soils’ (tissues, organs) (Pantel and Alix-Panabières, 2019). In many cases, successful metastatic colonization is achieved by clusters of CTCs rather than by individual tumor cells (Heeke et al., 2019). While these clusters represent a minority of the CTCs found in the bloodstream, they have been shown to have a metastatic potential 23–50 times greater than that of their single-cell counterparts (Aceto et al., 2014). Moreover, the presence and size of CTC clusters are associated with worse clinical outcome when compared to single CTCs in multiple cancer types (Murlidhar et al., 2017).

Box 2. Examples of GPC in cancer: CTCs.

CTC clusters are defined as groups of two or more CTCs that have stable cell–cell linkages and travel together through the bloodstream (Castro-Giner and Aceto, 2020). Aceto and others reported that CTC clusters have a higher potential for metastasis compared to individual CTCs, suggesting that group dynamics is an important component of metastatic success, at least for a cell’s capacity to initiate metastasis (Castro-Giner and Aceto, 2020). Even though characterization of CTC clusters is still in its infancy, different studies have suggested that these groups of cells collectively comprise key and complementary biological features that provide the clustering cells with novel characteristics compared to single CTCs.

For instance, the presence of clusters as well as their size are correlated with a poorer clinical outcome compared to the presence of single CTCs in cancer patients (Aceto et al., 2014). Interestingly, CTC clusters in breast cancer initially detach as multicellular aggregates from the primary tumor, precluding the occurrence of intravascular CTC aggregation (Aceto et al., 2014; Cheung et al., 2016).

A recent study might explain the advantage of clustered CTCs. In response to detachment from the extracellular matrix, reactive oxygen species are produced. The cell clusters could be protected from this phenomenon by the production of Hif1α and, subsequently, by a metabolic switch to glycolysis as well as increased survival and metastatic capacity (Labuschagne et al., 2019). This in vitro study remains to be validated in human CTC clusters under physiological conditions. Additional data demonstrated that CTC clusters show different gene expression profiles and dissemination modes compared to single CTCs. Indeed, the epithelial features (cell–cell adhesion via plakoglobin, keratin 14, and E-cadherin) observed in CTC clusters may be important in their formation and maintenance in the circulatory system and may also promote their collective migration and survival beyond maintaining contact between cells (Padmanaban et al., 2019).

Epigenetics also seems to play a key role since DNA methylation is clearly different between single CTCs and CTC clusters. CTC aggregation results in the hypomethylation of binding sites for stemness and proliferation regulators (e.g., OCT4, NANOG, SOX2, and SIN3A). Interestingly, this methylation profile is reversible by dissociating CTC clusters into single cells (Gkountela et al., 2019).

CTC cluster dissemination might also have an impact on a tumor’s developmental dynamics in the metastatic setting. In fact, heterogeneous clusters might seed polyclonal metastases, suggesting that this modality of cancer spread may increase the likelihood that a tumor will colonize distant sites successfully and might eventually show increased resistance to anti-cancer therapies (Castro-Giner and Aceto, 2020).

CTCs can form heterotypic interactions with non-tumor cells such as immune cells and cancer-associated fibroblasts (Giuliano et al., 2018; Herath et al., 2020). Szczerba et al., 2019 reported that neutrophils are the most abundant type of immune cell accompanying CTCs; such heterotypic clusters enhance the metastatic potential of CTCs by boosting their proliferative abilities. Alternatively, CTCs can circulate by interacting with fibroblasts, favoring metastasis formation by ‘bringing their own soil’ (Duda et al., 2010). Moreover, Heeke et al., 2019 mentioned that the CTC clusters have a clear advantage because they ‘never travel alone’ and they have close crosstalk with the circulating microenvironment. Indeed, cancer-associated fibroblasts and CTCs are considered as ‘dangerous liaisons’ (Hurtado et al., 2020), since cancer-associated fibroblasts (CAFs) secrete different proteins (cytokines, chemokines, growth factors) that promote invasion and angiogenesis, induce migration of tumor cells, and even confer evasion of the immune system. In conclusion, different types of clusters exist; the very rare and most aggressive ones are the heterotypic CTC clusters compared to the homotypic clusters and single CTCs (Castro-Giner and Aceto, 2020; Duda et al., 2010). The GPC framework could eventually help decipher which CTC clusters are most likely to metastasize (and where). This would provide predictive and/or therapeutic opportunities regarding metastatic success.

 CTC clusters can consist of similar or different tumor cells (e.g., expressing epithelial or mesenchymal markers). Clusters can also include non-tumor cells (e.g., blood cells, immune cells, and/or stromal cells) that can affect the fitness of cancer cells (Szczerba et al., 2019). According to our terminology, the single CTCs and CTC clusters composed of homogeneous cells would correspond to the simplest expression of a subgroup that, if successful, would proliferate and diversify to create larger groups with new specific GPCs. On the other hand, CTC clusters consisting of different cells can be viewed as small groups with a GPC that can influence their success during the dispersal, dissemination, and colonization steps. Importantly, the GPC of metastasis resulting from CTC clusters can also change over time, and these changes might be essential for the success of metastatic colonization and progression. For instance, dynamic changes in the epithelial and mesenchymal composition of CTC clusters in breast cancer have been associated with disease progression (Yu et al., 2013). Nevertheless, it is less clear whether this heterogeneity reflects potential cooperative interactions among cells within a CTC cluster and whether this change in GPC impacts metastatic efficiency. The extent to which CTC cluster composition should recapitulate the parental GPC (perhaps with modifications) to be successful remains to be determined. We cannot exclude that specific GPCs, different from the parental one are required to target given organs, with convergence between metastastic tumors independent of the localization of their primary tumors (Cunningham et al., 2015). Box 2 discusses CTCs as an example of the GPC dynamics in cancer.

### GPC in transmissible cancers

The GPC concept can also be extended to transmissible cancers (see also Box 3 that uses transmissible cancers as another example of the GPC in cancer). Previous work has argued that the emergence of transmissible cancers required a ‘perfect storm’ with the convergence of multiple host (micro- and macroenvironmental factors) and tumor cell traits (Ujvari et al., 2016a). Here, we hypothesize that the tumoral GPC is also an important component of the ‘perfect storm’ necessary for the evolution of a transmissible cancer. The majority of cancer cells that emerge in multicellular organisms die with their host, but there are at least nine well-described, independent exceptions of transmissible cancers in the wild: one in dogs (called canine transmissible venereal tumor, CTVT), two in Tasmanian devils (called devil facial tumor diseases, DFTD and DFT2), and six in six bivalve species (called bivalve transmissible neoplasias [BTN], see Ujvari et al., 2017; Yonemitsu et al., 2019). In these diseases, malignant lineages behave like infectious agents, with cancer cells spreading horizontally as allografts and/or xenografts from one individual to conspecifics, or to individuals of (closely) related species. These cancer cells are currently viewed as new parasitic species, with their own evolution and dynamics (Dingli and Nowak, 2006).

Box 3. Examples of GPC in cancer: transmissible cancers.

Compared to human cancer research, the study of transmissible cancers is in its relative infancy, with fewer and smaller teams working on the topic (Dujon et al., 2020b). Finding empirical evidence for GPC to exist in these (so far) rare cancers (Dujon et al., 2021) is less straightforward. Nevertheless, based on emerging transcriptomic, genomic, and experimental data, predictions could be made for their existence. For example, experimental transmission of all three types of transmissible cancers indicates the potential relevance of GPC. Immunocompromised xenograft murine models have been developed for both DFTD (Kreiss et al., 2011) and CTVT (Holmes, 1981; Harmelin et al., 2001). In the DFTD model, NOD/SCID mice inoculated with either 10^5^ or 10^6^ viable DFTD tumor cells developed tumors after 7 and 4 weeks, respectively, and X–DFTD tumors reinoculated into NOD/SCID mice formed visible tumors after 5 weeks (Kreiss et al., 2011). In CTVT murine models, 10^6^ cells were sufficient to cause tumor development in NOD/SCID mice (Harmelin et al., 2001). A study from the early 1980s that followed the shading of radioisotope-labeled CTVT cells in urine found that after the initial rapid phase of cell death, approximately 10% of the injected label was detectable, and subcutaneous CTVT nodules developed by day 7 (Holmes, 1981). Similarly, in the transmissible cancer system of bivalves, experimental transmission of 10^6^ BTN cells induced cancer in 33% of new hosts, and transfection of 10^7^ BTN cells resulted in a 50% infection rate (House, 1997b). These studies indicate that although most likely a large proportion of cancer cells die during transmission, a small population of viable cells could be sufficient to establish a tumor mass. While there is currently no evidence available, it could be proposed that—similar to CTCs—the success and survival of transmissible cancer cells may be higher when ‘traveling’ in groups or having a particular GPC at a given time point of transmission (e.g., extravasation, transmission, intravasation). Collective transmission of a group of cells with diverse and/or particular phenotypes may modulate the emergent properties of transmissible cancer cells that facilitate the breakdown of tissues as well as individual and environmental barriers, eventually leading to persistent group-level differences in fitness. This could potentially be most relevant for transmissible cancers in bivalves, where transmission occurs by release and uptake of hemocytes into and from the aquatic environment (Metzger et al., 2016). Disseminated neoplasia cells have been shown to have relatively broad tolerance to environmental conditions (10–15% mortality below 20°C that rapidly increases to 80% at 30°C, salinity tolerance between 0.5 psu and <35 psu, and pH preference between pH 4 and 9.3 [Bramwell et al., 2021; Sunila, 1991]); this plasticity might be the result of and facilitated by the production of different GPC released by BTN cells at a given point in time.

Although empirical evidence is currently lacking to support the hypotheses outlined above, future transfection experiments could prove or disprove these theories. For example, mussels have a natural tendency to open their valves and to release and uptake hemocytes (Caza et al., 2020). Placing mussels under different environmental conditions and exposing them to stained BTN cells (e.g., CFSE, a cytosol dye commonly used for tracking cells in vivo by flow cytometry), followed by transcriptome and epitome analyses of both established BTN and host cells may reveal the phenotypic composition of BTN cells that were successfully taken up by and survived in the new host (Caza et al., 2020).

Another direction for investigating the importance of GPC in transmissible cancers could be the Tasmanian devil and its two independently emerged contagious cell lineages. The first lineage, DFT1, was initially observed in 1996 in the northeastern corner of Tasmania; it has quickly spread across Tasmania, including the southern regions. The second independently risen lineage, DFT2, was discovered in 2014 (McCallum et al., 2009; Pye et al., 2016) in the southern corner of Tasmania. The distributions of the two lineages now overlap in the d’Entrecasteaux Peninsula (James et al., 2019). DFT1 tumors mostly occur on the face, with non-facial tumors more commonly found in DFT2 (130), indicating potential niche partitioning of the host by the two cancer lineages. Although co-infection by DFT1 and DFT2 occurs, it has been rare thus far, and DFT2 has seemingly started replacing DFT1 on the local scale (James et al., 2019). The current rapid expansion of the DFT2 lineage may be occurring because its high levels of GPC facilitate evasion of immune recognition. As described above, the two lineages employ different immune system evasion strategies: DFT1 cells lack MHC expression on their cell surface while DFT2 cells express classical and non-classical MHC alleles (Caldwell et al., 2018). The expression of MHC molecules in DFT2 is not uniform – it is highly heterogenic in vitro and in vivo. For example, some DFT2 and DFT1 cells share MHC class I alleles, some DFT2 cells also express high levels of an MHC class I allele common to its hosts, and some DFT2 cells do not express MHC at all (Caldwell et al., 2018). Although only conjectural, it is possible that during transmission of a cluster of tumor cells, the heterogeneity in immune recognition evasion may be beneficial for these contagious cancers since it may affect phenotype-dependent predation risk via confusion and oddity effects (i.e., a complete lack of MHC expression could trigger natural killer cell responses, but these are being silenced due to certain cells expressing alleles similar to the host).

While conducting co-infection experiments on Tasmanian devils would be nearly impossible, in vitro cell competition assays could potentially be employed to investigate whether the GPC of the two lineages and co-infection by ‘mixed-species’ (the two cell lines representing species in this case) would benefit one or both lineages via resource allocation and niche partitioning.

The first steps during the emergence of a transmissible cancer are likely to be similar to non-transmissible ones (i.e., as described in the sections above), with similar constraints on the GPC composition required for a tumor to grow and metastasize. However, to become transmissible, a cancer must also gain the ability to shed a very large number of cells that can both survive in the environment outside the host’s body (Sunila and Farley, 1989), but also be able to infect new hosts (Ujvari et al., 2016b). Since we have thus far only observed transmissible cancers that successfully spread to a large number of individuals, there is at the moment no evidence of what would be a favorable tumoral GPC for an emerging cancer to become transmissible (at least for the infectious stage). Both CTVT and DFTD manifest as undifferentiated cancers, showing strikingly uniform cell morphology. However, the tumors are not completely homogeneous, and variability in antigen expression in and between tumors has been observed (O'Neill, 2011). Since they are found in both host species, those tumoral GPC characteristics are likely to be important ones for a cancer to be transmissible.

Once inside the body of a new host, most transmissible cancer cells fail to establish a new tumor (10^5^–10^7^ cells were required in transmission experiments [House, 1997a; Kreiss et al., 2011]). This high failure rate can be partly explained by the fact that a large proportion of cells are eliminated by the host’s immune system or fail to find a suitable environment to establish. In addition, it is also possible that most of the newly initiated transmissible cancer tumors regress because of a tumoral GPC mismatch (in a similar way many of the de novo tumors can fail) (see further details in Box 3).

It is also possible that different types of tumoral GPC are required for every step of the intra- and inter-individual transmission process. As transmissible cancers show intra- and inter-individual metastasis, every successful transmission requires establishment in a novel microenvironmental niche and the avoidance of different levels of immune recognitions. For example, a particular GPC may be beneficial when spreading within a host via initiating immune tolerance to the tumoral GPC, but the same GPC may also present as additional immune target, and thus not be ‘optimum’, when transmitting across hosts. For example, the newly emerged devil facial tumor disease, DFT2, avoids immune recognition by various mechanisms: some tumors express MHC class I molecules that are shared with host devils, some express non-classical MHC molecules, while in some*﻿* MHC class I, expression has been completely lost (Caldwell et al., 2018). Further work would be needed to determine whether this also occurs within tumors and to establish the extent of intratumoral variability in these features.

A major difference between non-communicable cancers and transmissible cancers is that classical cancer cells have to ‘reinvent the wheel’ at every step – from initiation and progression to metastasis, but ultimately, they all die with the death of the host. In contrast, in transmissible cancers, clonal cell lineages are passed from host to host, and thus evolve as novel parasitic life forms, as seen in CTVT and DFTD, where these cancer lineages have continued to evolve since their initial emergence (Pearse et al., 2012; Dujon et al., 2020a; Kwon et al., 2020). Thus, investigating whether the tumoral GPC has been adjusted by selection in a way that optimizes the parasitic lifestyle will also provide insight into the evolution of parasitism. Focus on BTN, where host switch has been observed, would be particularly interesting as different tumoral GPCs might have been required at every stage of transmission (within host, between hosts, and between species). There are, however, no data available concerning the GPC of transmissible cancers, especially at infection time. Therefore, all the hypotheses discussed above remain to be tested.

## Therapeutic implications

Currently, the major focus of therapeutic development is on directly targeting viability or proliferation of tumor cells. Consideration of the GPC perspective could suggest new therapeutic approaches to achieve desirable clinical outcomes. While, at this point, prerequisite knowledge is still lacking, below we provide several suggestions on the new therapeutic angles inspired from the GPC framework.

First, we should attempt to identify keystone tumor cell subgroups/phenotypes – those critical for the maintenance of tumors and transitions (i.e., those that contribute to the ‘optimum’ GPC, Figure 2) at key steps of their progression (invasion, metastasis, acquisition of therapy resistance), and either target them directly or focus on the mechanisms underlying cross talk among phenotypes. This strategy, compared to those that target the entire heterogeneous tumor, will be more efficient because it would target smaller and more homogenous and less diverse subgroups. This should decrease the risk of emergence of resistant clones. The emergence of therapy resistance is also accompanied by changes in GPC; understanding the specific GPCs that promote resistance should help develop strategies that prevent the acquisition of resistant GPCs. Below we expand on these two complementary approaches and provide specific experimental directions.

In order to gain the ability to consider GPC in therapeutic decision making, we first need to develop approaches to identify keystone subgroups/phenotypes and specific combinations of interacting phenotypes that result in optimum GPCs. Examples of such keystone subgroups and interactions have been identified in multiple experimental contexts (Tabassum and Polyak, 2015). For example, in experimental models of breast cancer heterogeneity, interleukin 11 expressing subpopulations can be responsible for driving tumor growth and metastatic dissemination via mechanisms involving microenvironmental changes (Marusyk et al., 2014). Similarly, aggressive subtypes in glioma and breast cancer can affect, via paracrine signaling, the phenotype and metastatic potential of less aggressive subtypes (Neelakantan et al., 2017; Espinoza-Sánchez et al., 2017). However, transitioning from proof of principle studies toward characterizing GPC in primary tumors and identifying key subpopulations would necessitate stepping out of the dominant focus on molecular mechanisms, following ‘Gene X is a critical regulator of hallmark H’ algorithm, and developing system-level understanding of tumor cell subpopulations and their interactions. Recent advances in single-cell analyses, such as single-cell RNA sequencing and high multiplexing histological analyses, are already providing us with detailed characterization of phenotypes of individual tumor cells and/or subgroups that constitute the GPC. However, in order to transition from simply describing the GPC into identification of keystone subgroups and key biological interactions, we would need to develop network analysis pipelines that explicitly focus on the analyses of expression of receptors and corresponding ligands (including both secreted ligands and extracellular matrix (ECM)), and identify or engineer experimental systems that recapitulate individual interaction nodes, enabling the interrogation of the consequences of their disruption. Moreover, rather than focusing on a single interaction at a time, we will need to develop strategies for co-disrupting redundant interactions responsible for robustness and antifragility of tumor ecosystems.

Second, in many cases of systemic treatment of multisite metastatic disease, individual tumors display marked variability in responses to therapy, ranging from complete elimination to lack of detectable therapeutic responses (e.g., Patel et al., 2021). Comparative analyses of the GPC of ‘successful’ versus ‘failed’ tumors represent another potential approach to interrogate the role of GPC in the development of resistance to therapies. While, traditionally, the explanation sought is in the identification of cell-intrinsic ‘drivers’ of resistance, comprehensive single-cell characterization of pre-treatment biopsies could discriminate between GPCs associated with tumors that succumb to therapy and those that develop resistance. Even if metastases are rarely resected, which hampers their characterization at the cellular/molecular level, such analyses might sometimes be feasible in clinical presentations of multiple skin metastases, such as in Wagle et al., 2011, or in animal models of heterogeneous multi-metastatic disease. A limitation to this suggestion is, however, the fact that certain cells and/or subgroups important for the GPC could be missed for technical reasons.

Third, the phenotypic composition of successful/resistant tumors is also influenced by the ability of tumor cells to switch phenotypes. Therefore, explicitly targeting phenotypic plasticity (independently of cytotoxic/cytostatic effects of treatment) might be able to prevent plastic adaptive changes in GPC and improve therapeutic outcomes. Phenotypic plasticity should not be considered strictly as a means to increase individual/cell-level fitness. In our framework, phenotypic plasticity is also a way for cancer cells to both rapidly change the tumoral GPC to maintain the combination of phenotypes required for progression or overcome the therapeutic suppression of a dominant aggressive phenotype. This means that optimal or suboptimal tumoral GPCs could be restored after such interventions simply as a consequence of these non-genetic events. To circumvent these phenomena, targeting phenotypic plasticity should be relevant, either alone or in combination with targeted therapies (Doherty et al., 2016). This is essential when considering combinations with other therapies targeting one or several particular phenotype(s)/subgroup(s) because alteration of the possibility to change the tumoral GPC should avoid reconstituting the missing subpopulation.

Phenotypic plasticity is extremely relevant especially during the epithelial-to-mesenchymal transition (EMT). EMT generates mesenchymal-like cells and a variety of intermediate cell states between the epithelial and the mesenchymal state and changes the GPC toward tumor progression and metastasis (Brooks et al., 2015; Dongre and Weinberg, 2019; Rios et al., 2019). Thus, targeting molecular machinery involved in phenotypic rewiring (such as inhibitors of HDAC, KDM, BETs) should limit the evolution of the GPC both during tumor growth and after a therapeutic intervention. Many other molecular mechanisms underlie cell plasticity, for instance the Notch and Wnt development pathways, or those involving transcription factors from the Snail, Zeb, and Twist families, whose role in the generation of plasticity and cell-to-cell heterogeneity in cancer partially overlaps with their role during development and wound healing (Gupta et al., 2019). Deregulation of these transcriptional regulators may allow cancer cells to access resistant phenotypes by rewiring of gene regulatory networks (Marusyk et al., 2020).

More globally, insights can be gained on the role of phenotypic plasticity in affecting tumor GPC using dynamical systems theory (Huang et al., 2009; Jia et al., 2017) where reshaping of the epigenetic landscape in cancer cells allows acquisition of novel abnormal cell states corresponding to ‘cancer attractors’ in the gene regulatory network (Huang et al., 2009). The enhanced cellular stochasticity in cancer cells would facilitate these phenotypic conversions toward novel states (Huang et al., 2009; Li et al., 2016). Stochasticity of gene expression appears to be increased in cancer cells (Jenkinson et al., 2017), and diverging chromatin states and transcriptional heterogeneity produce corrupted coordination of epigenetic modifications (Pastore et al., 2019). Also, the stochastic nature of gene expression fosters a transient resistant phenotype by chance, which can then be stabilized by epigenetic mechanisms (Shaffer et al., 2017). As such, modulating stochasticity, especially through tuning of gene expression variability, might represent a good strategy to control tumoral GPC evolution (reduced phenotypic heterogeneity will limit cooperative interactions). The global epigenetic changes generally observed in cancer cell epigenomes could find their origin in ‘tumor reprogramming’ due to genetic alterations initiating cancer that would contribute to cancer growth less by inducing cell proliferation than by causing ‘developmental reprogramming’ of the epigenome and allowing cells to aberrantly and pathologically differentiate (Vicente-Dueñas et al., 2015; Xiong et al., 2019). Another possibility is that this global destabilization of gene expression could originate from tissue disruption-induced stochasticity (Capp, 2005; Capp, 2017; Capp, 2019; Capp and Bataille, 2020), suggesting that restoring cellular interactions present in the initial tissue for instance with molecules that would mimic healthy cellular interactions would reduce cell stochasticity and restabilize cellular phenotypes (Capp and Bataille, 2020; Capp, 2012; Capp and Bataille, 2018). In any case, disrupting the tumoral GPC will require innovative strategies based on the control of phenotypic plasticity.

Fourth, while currently relevant knowledge is almost entirely lacking, it will be important to understand whether and how the tumoral GPC is reestablished in a secondary tumor, and the role the tumoral GPC plays in metastatic colonization and outgrowth. We can speculate that since metastases can be seeded by individual cells or small groups of cells (Cheung and Ewald, 2016), the odds that the ‘right combination of cells’ is initially present will be very small. Consequently, if the optimum GPC needs to be reestablished at a metastatic site, the failure to do so could contribute (along with the many other hurdles faced by prospective metastases) to the extremely low colonization rate of circulating tumor cells. In addition, the establishment of a new tumor in a completely different tissue microenvironment (e.g., breast cancer metastasis to the bone) should require a GPC that is quite distinct from that in the native site. The growing metastatic tumor would need to adapt to a very different environment, which could be facilitated by phenotypes that secrete cytokines or chemokines that both recruit regional cells into the tumor’s service and impede antagonistic cells like cytotoxic T cells, by other phenotypes that promote degradation of the extracellular matrix and/or acidification, and so forth (Quail and Joyce, 2013). The assembly of such a GPC may (fortunately) represent a substantial hurdle to metastatic success. On the other hand, the seeding metastatic cells may already possess greater phenotypic plasticity and an enhanced capacity for generating genetic and epigenetic heterogeneities.

Fifth, in addition to targeting keystone subgroups, tackling specific interactions (such as clonal cooperation) may be of equal or higher therapeutic benefit due to altering tumoral GPC quality. For instance, disrupting clonal cooperation by targeting key factors was already suggested (Zhou et al., 2017), as well as their shared external products (i.e., ‘public goods’) (Pepper, 2012). A list of potential targets includes members of signaling pathways, such as the proto-oncogenes Wnt1 or Hedgehog, and microenvironment-related factors, such as matrix metalloproteinases (Zhou et al., 2017), although the exact useful target is likely to be specific to each individual tumor type. Inhibition of tumor progression can be achieved by disruption of these clonal cooperative interactions, which impair the tumor ecosystem and the tumoral GPC. While only a few of the many pathways/molecules involved in clonal cooperativity are characterized, it should be expected that the coming years will bring new opportunities to inhibit this aspect of tumor biology.

## Concluding remarks

Tumors can be seen as examples of social heterogeneous groupings. A proper mechanistic understanding of the impact of ITH and tumoral GPC on cancer progression requires bridging oncological sciences with evolutionary ecology. What are the potential advantages of GPC consideration over traditional, reductionistic approach of viewing tumors as collection of specific genetic clones or phenotypic subpopulations? A first obvious benefit of the GPC concept resides in the fact that it permits assessment of some of the functional aspects of cancer dynamics, which are impossible to understand by simply examining cellular collections only. In the same way that we cannot fully understand the biology of any living organisms when examined in isolation from its abiotic and biotic environment (including its conspecific peers), the biology of malignant cells must consider the social context (i.e., the GPC) to which it is exposed. The GPC concept should also permit us to understand phenotypic convergences, when cells differ at the genetic or epigenetic level but accomplish more or less the same tasks (see Box 1).

On the other hand, the utility of considering tumor GPC is currently limited by the lack of criteria to define relevant groups of tumor cells. Strong interdisciplinary research that integrates observational, experimental, and theoretical studies will be crucial to develop methodology to define and categorize GPCs, identify functionally relevant subpopulations within heterogeneous tumors, identify critical interactions between these subpopulations, and identify the parameter values of GPC descriptors that are associated with an ‘optimum’ GPC at each cancer progression step. Such studies will ultimately allow us to understand the impact of GPCs (and the interactions between metastable components of the GPCs and their microenvironments) on the collective behavior of tumors and their evolutionary and clinical trajectories.

In this paper, we did not consider the potential existence of metaphenotypes of structured groups of cells, that is functional clusters, within tumors. Such a situation would yield to different and more complex GPC-related dynamics on tumor progression. While whole tumors are (most often) not in competition within tissues, this can conversely be the case for functional clusters within tumors, suggesting that the GPC of tumors could display a ‘nested structure’. That is, the GPCs of functional clusters within the tumor are also influenced by the competition between groups, but these groups also contribute together to a whole tumoral GPC that is not in a competitive context with other tumors. In addition, if a group within the tumor has a better GPC than others, it can increase in prevalence, with tumor growth then seeding the formation of new GPCs. More research is needed at the moment to explore the extent to which tumor dynamics are influenced by the whole tumor GPC, by GPCs of functional groups that are within tumors, and/or by a mix of both. Also, we need to determine whether there are temporal patterns in these processes, exploring for instance the importance of these different dynamics throughout tumorigenesis. Still, the formation of ‘the right GPC’, appearing in the right condition at the right time, is heavily influenced by chance, stochasticity and spatial context, and thus remains challenging to investigate and act on. For instance, cells that would form a viable GPC for host-to-host transmission may exist but are not co-localizing or cannot be transmitted as a unit during shedding/infection. Similarly, some phenotypes may benefit from an advantage simply because they are close to angiogenic cells, while other GPC-formation candidates in other parts of the tumor will die due to nutrient scarcity. Other information is also needed to determine whether the propensity for developing the ‘right’ GPC (see Figure 2) during tumorigenesis may rely on the individual’s genetic predisposition. It is noteworthy that even for genetic predisposition syndromes that confer the highest probability of developing cancer such as those resulting from inherited dysfunction of BRCA1/2 (predisposing to breast and ovarian cancers) and mismatch repair genes (e.g., Lynch syndrome), a significant proportion of patients (30–40%) do not develop cancer by the age of 70 (Antoniou and Easton, 2006). The GPC might well be an important modulating factor of the risk over time.

Overall, examining tumor GPC and addressing the effects of GPC on individual tumor cell fitness (selective consequences) and the responses of individual cells to the selective consequences of GPC (evolutionary consequences) should improve our understanding of tumor biology and cancer progression. At the moment, it remains mostly an abstract concept because we need to develop novel tools to describe, understand, and monitor the tumoral GPC functional dynamics and its resilience to local disturbances. Also, criteria used to describe groups remain by definition subjective, limiting the possibility at the moment to provide a unique definition of GPC in cancers (see Box 1 for a tentative example). This knowledge will also need to move beyond this theoretical phase to become useful for diagnostics or therapeutic design, providing opportunities for therapies that will specifically and durably disrupt key components and interactions within tumors.
